# In a Randomized, Placebo-Controlled Cross-Over Study, Administration of 6 and 12 G Fortetropin® Does Not Reduce Serum Myostatin in Healthy Adult Dogs Over 72-Hours

**DOI:** 10.3389/fvets.2021.680576

**Published:** 2021-07-22

**Authors:** Carmella C. Nugent Britt, Leilani X. Alvarez, Kenneth Lamb

**Affiliations:** ^1^The Animal Medical Center, New York, NY, United States; ^2^Lamb Consulting, West St. Paul, MN, United States

**Keywords:** biomarker, sarcopenia, muscle atrophy, myostatin, Fortetropin, growth differentiation factor 8

## Abstract

**Objective:** To evaluate the effect of a single administration of 6 and 12 g of Fortetropin compared to placebo on serum myostatin in healthy, adult dogs over a 72-h period.

**Methods:** Prospective, placebo-controlled, randomized, double-blind, crossover study. Ten hospital-employee-owned healthy adult dogs aged 2 to 8 years old were enrolled in the study. Blood samples were collected prior to and then 12-, 24-, 36-, 48-, and 72-h following administration of the test agent (6 and 12 g) or placebo. Serum samples were processed according to manufacturer's guidelines for canine serum using GDF-8/Myostatin Quantikine ELISA kit (R&D Systems). Analysis-of-variance (ANOVA) analyses were carried out where *P* < 0.05 was deemed significant.

**Results:** Mean serum myostatin was not significantly lower in treatment groups of either low or high dose compared to placebo at any time point. Baseline mean serum myostatin in low and high dose treatment groups was 29,481 (SD = 5,224) and 32,214 pg/mL (SD = 7,353), respectively. Placebo group low and high dose baseline mean serum myostatin was 30,247 (SD = 5,875) and 28,512 (SD = 5,028).

**Conclusion:** The results of this study indicate that administration of single 6 or 12 g dose of Fortetropin does not reduce serum myostatin in healthy adult dogs over a 72-h period.

**Clinical Importance:** Oral supplements, like Fortetropin, require further studies to determine the efficacy and bioavailability in order to guide clinical use in dogs.

## Introduction

Muscle mass is determined by multiple factors including genetics, hormones, age, nutrition, disease, injury, and activity (1). Myogenic differentiation is further regulated through complex signaling pathways involving multiple cytokines and growth factors (2, 3). Myostatin, also known as growth differentiation factor 8 (GDF-8), is a member of the transforming growth factor β superfamily and plays a significant role in negative regulation of muscle mass. It may also play a role in promoting muscle atrophy (2, 3).

Myostatin negatively regulates muscle mass by promoting increased protein degradation downstream through the ubiquitin-proteasome pathway (1, 4). Furthermore, myostatin maintains a dormant population of satellite cells, thereby inhibiting myoblasts from proliferation (5–7). Myostatin can exist in two forms: latent and biologically active. Latent myostatin circulates in plasma bound to several proteins (8). To become biologically active, myostatin is cleaved twice in response to upstream mediators of the signaling cascade (9). Baseline myostatin and normal fluctuations of myostatin are not known in dogs.

Mutations of the myostatin gene, leading to a myostatin deficiency as seen in double-muscled Belgian Blue and Piedmontese cattle, knockout mice, and “bully” whippets, result in profound muscle hypertrophy (2, 10, 11). Resistance training and exercise have been associated with decreased myostatin (4, 11). In contrast, increased serum myostatin and myostatin mRNA are associated with periods of inactivity, sarcopenia, and cachexia of chronic disease (11, 12). As such, myostatin has been a heavily investigated target for potential therapies to treat or prevent muscle atrophy in the face of disease, injury, and disuse. One such therapy purported to inhibit myostatin in dogs is an oral supplement, Fortetropin® (Myos Corp).

Fortetropin, a proteo-lipid bioactive compound, is made from non-thermal pasteurized fertilized egg yolk (13). The exact mechanism of action of Fortetropin on myostatin has not been completely elucidated; however, early research suggests that it works through upregulation of the muscle hypertrophy pathway mTOR and decreasing markers for protein break down (4).

A study by Sharp et al. evaluating Fortetropin supplementation showed promising results in both myostatin inhibition and muscle hypertrophy (4). This double-blind placebo-controlled study evaluated rats receiving Fortetropin given once daily for 8 days combined with acute resistance training compared to those receiving a placebo. In this same study, the researchers also evaluated humans who received two doses of Fortetropin compared to a placebo. Expression of myostatin mRNA was significantly lower in groups that received Fortetropin (4). A small study in older men and women demonstrated that 21 days of daily consumption of Fortetropin increased fractional synthetic rate of protein by 18% compared to placebo (14). The White et al. study showed stable serum myostatin concentrations in dogs receiving daily Fortetropin supplementation for 12 weeks following a tibial plateau leveling osteotomy (TPLO). Dogs in the placebo group of this study experienced a significant rise in serum myostatin as well as a significant reduction in thigh circumference (13). Based on the evidence in this early literature, Fortetropin has potential to be an important supplement in dogs for the treatment or prevention of muscle atrophy from disuse, injury, sarcopenia, and cachexia.

In contrast to pharmaceutical agents, oral supplements often lack evidence supporting bioavailability, pharmacodynamics, and pharmacokinetics. The bioavailability, pharmacokinetics, and mechanism of action of Fortetropin has not been assessed in dogs. The aim of this study was to evaluate the effect of the administration of two different doses of Fortetropin (6 and 12 g) on inhibition of myostatin compared to a nutrient-matched placebo over a 72-h period in healthy adult dogs. We hypothesized that serum myostatin concentrations would have a dose-dependent inhibition in response to Fortetropin. A secondary aim was to evaluate the effect of age on serum myostatin and establish normal baseline myostatin concentrations over a 72-h period in healthy adult dogs.

## Materials and Methods

### Dogs

A total of 12 hospital-employee-owned dogs were enrolled in this prospective, double-blind, randomized, placebo-controlled study. Two dogs were dropped from the study prior to completing the first sequence, resulting in a total of 10 enrolled dogs. The protocol was approved by the Institution's Animal Care and Use Committee. Informed consent was obtained from each owner prior to the start of the study.

### Inclusion and Exclusion Criteria

Clinically healthy adult dogs (aged 1 to 8 years old) were considered for enrollment. A complete general, orthopedic, and neurological exam was performed on each dog by either a diplomate or resident in sports medicine and rehabilitation. All dogs had a screening complete blood count and blood chemistry, collected via peripheral venipuncture, and a free-catch urinalysis prior to enrollment. Dogs were excluded if any abnormalities on the prescreening laboratory work or exam were considered clinically significant. All dogs enrolled were within the preset weight criteria of 11.0 to 22.7 kg and a BCS of no <4 and no more than 6.

Subjects were free of any oral supplementation for 3 months prior to the beginning of the trial. Dogs had no history of being treated with pharmaceutical drugs that could interfere with muscle function (steroids, muscle relaxers, or chemotherapeutics). Diet and activity remained stable during the study.

### Novel Supplement

Single-dose vials of the placebo and the commercially available novel supplement, Fortetropin, were provided by Myos (Myos Rens Technology, Cedar Knolls, New Jersey) prior to the start of the study. The placebo was a cheese powder equivalent in macronutrient profile to that of the novel supplement (Protein 33 g, Carbohydrates 7 g, Fat 20 g). No third-party laboratory evaluation was performed to confirm the macronutrient profiles. Both a low (6 g) and a high (12 g) dose of the novel supplement and the equivalent placebo were tested. The low dose was determined by the manufacturer's recommended dose for dogs in our preset weight criteria and the high dose reflects double the recommended dose.

### Dosing and Sample Collection

Prior to the start of this study, pilot study was conducted to optimize dose and sampling methods. A single, female spayed, healthy, adult dog weighing 23 kg was given one 6 g dose of Fortetropin mixed in a small amount of food. Blood samples were collected 2-, 4-, 6-, 12-, 18-, 24-, 36- and 48-h following administration of the test agent. Data from this unpublished study showed that a single dose of 6 g of Fortetropin given reduced serum myostatin concentrations by almost 50 percent with a trough at 36-h. This data was used to develop the current study protocol.

**Figure 1:**
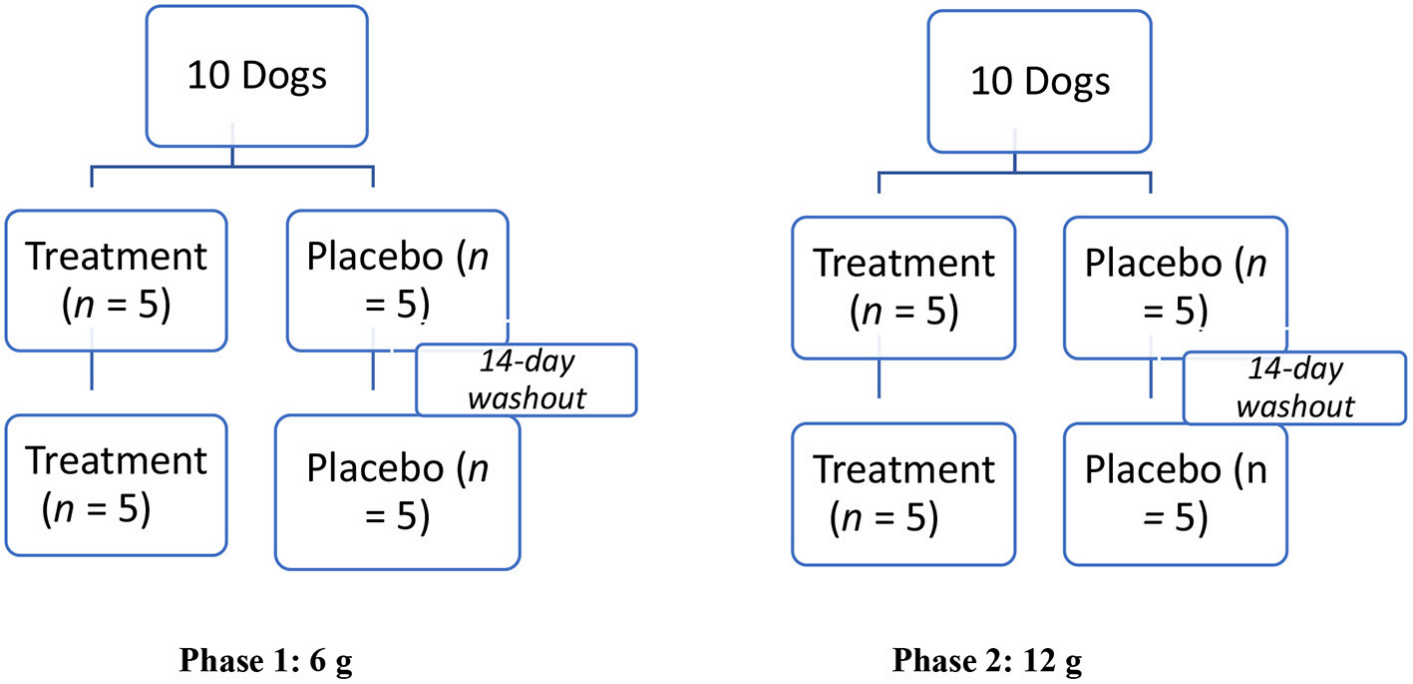
Modified crossover experimental design testing effect of a muscle supplement, Fortetropin®, on serum myostatin in 10 healthy dogs. Two phases, each with two sequences of the study, were performed. In Phase 1, 5 dogs were randomly assigned to receive a single low-dose (6 g) treatment or placebo for the first sequence. After a 14-day washout period, the animals were re-randomized into placebo and the treatment groups. This same protocol was repeated in Phase 2 for the high-dose (12 g) treatment or placebo.

A modified crossover design was used for dosing (Figure 1). The low (6 g) and high (12 g) doses of the supplement were compared to equivalent amounts of the placebo in two phases (Figure 1). In the first phase, the low dose was evaluated, and in the second phase, the high dose was evaluated. Each phase consisted of two sequences. In the first sequence of each phase, dogs were randomly assigned to one of two groups (treatment, *n* = 5; placebo, *n* = 5) using an online randomizing program (GraphPad Quick Calcs). In the second sequence of each phase, dogs were re-randomized to one of two groups (treatment, *n* = 5; placebo, *n* = 5). After completing each phase (total of four sequences), a total of 10 dogs were enrolled in the low (6 g) and high (12 g) novel supplement groups, and 10 were enrolled in each placebo (6 and 12 g) group, respectively.

On day 1 of each sequence, a single dose of either the placebo or novel supplement was administered by a blinded research assistant. The test agents were labeled A (novel supplement) or B (placebo) to maintain blinding. The test agent was mixed into ¼ cup of Hills® (Topeka, Kansas) Prescription Diet® i/d Canine canned food and fed to the dog. The assistant confirmed that the entire sample was consumed by the dog. There was a 14-day washout period between each sequence and each phase to ensure clearance of the test agent.

Blood samples were collected at time 0 (prior to administration of the test agent or placebo), and 12-, 24-, 36-, 48-, and 72-h after administration of the test agent or placebo. Approximately 1 cc of blood was collected via jugular venipuncture using a 3 cc syringe and a 22 G needle. The samples were collected into serum separator tubes (Becton Dickinson, Franklin Lakes, New Jersey). The tubes clotted at room temperature for 2-h before they were spun for 30 min at 2,000 rpm. The serum was then aliquoted in triplicate (~200 μL per vial) into cryovials (Millipore Sigma, Darmstadt, Germany) and stored at −80°C until they were processed. Preparation of all serum samples was performed by a single person), who was blinded to the test agent given to the dogs.

### Myostatin ELISA

The frozen serum samples were shipped on dry ice in duplicate to Kansas State University for processing. The laboratory personnel were kept blinded to the test agent given to the dogs. All samples were processed on the Quantikine© ELISA GDF-8/Myostatin Immunoassay (R&D Systems, Inc., Minneapolis, Minnesota, Catalog # DGDF80) according to the manufacturer's guidelines (8). Briefly, 20 μL of serum was treated with 10 μL of 1 N hydrogen chloride and incubated at room temperature for 10 min. An additional 10 μL of 1.2 N sodium hydroxide/0.5 M HEPES was then added to the sample and mixed well. Finally, 560 μL of the provided calibrator diluent RD5-26 was added, and the sample was mixed. The final solution resulted in a 1:30 dilution of serum.

A 96-well plate (R&D Systems, Inc., Minneapolis, Minnesota) was used for the ELISA. In each well, 50 μL of assay diluent RD1-17 (R&D Systems, Inc., Minneapolis, Minnesota) and 50 μL of either standard, control, or sample were added. This was incubated for 2-h at room temperature on a horizontal orbital microplate shaker set at 500 rpm. After 2-h, the wash was performed a total of 4 times with the wash buffer provided with the kit (R&D Systems, Inc., Minneapolis, Minnesota). After the buffer wash, 200 μL of the GDF-8 conjugate was added to each well and incubated at room temperature for 2-h. The buffer was repeated, and then 200 μL of substrate solution (R&D Systems, Inc., Minneapolis, Minnesota) was added to each well. After 30 min at room temperature on the bench top, 50 μL of the stop solution was added to each well. The optical density of each plate was then measured using a microplate reader set to 450 nm.

### Statistics

Descriptive statistics were calculated for all groups at all time points. Between-group and within-group analyses were performed using independent and repeated measures dependent model analysis of variance (ANOVA), as error residuals were normally distributed. The reduced model of main effects treatment and time coupled with interaction was employed omitting sequence and nested effect patient within sequence as the washout effect was non-significant. The ANOVA was used to compare baseline myostatin serum concentrations. All analyses were carried out using SAS 9.4 statistical software (Cary, North Carolina 2013) where *P* < 0.05 was deemed significant. A power analysis was not performed prior to conducting the study.

## Results

### Study Population

Of the 12 dogs enrolled, 10 completed the study. One dog was withdrawn from the study after vomiting the test agent within 12-h of administration. A second dog was withdrawn from the study after abnormal bruising occurred during venipuncture and was subsequently diagnosed with a clotting disorder not detected in routine screening.

The enrolled dogs ranged in age from 2 to 8 years. The median and mean ages were 4.0 and 4.2, respectively. There were 9 spayed females and 1 intact male. Breeds included Golden Retriever (*n* = 1), Border Collie (*n* = 2), Staffordshire Terrier (*n* = 1), and mixed breeds (*n* = 6). Body condition scores (BCSs) ranged from 4 to 6 out of 9 with a median of 4/9 and a mean of 4.8/9.0. Weights ranged from 14.3 to 22.4 kg with a median of 19.85 and a mean of 19.4 kg.

### Side Effects

A few dogs experienced minor side effects after administration of the test agent and the placebo. Two dogs in the treatment group had a single episode of soft but formed stool approximately 48-h after administration. Two dogs in the placebo group experienced multiple bouts of diarrhea through the duration of a sequence that resolved without intervention.

### Serum Myostatin

#### Low-Dose Fortetropin Compared to Low Dose Placebo

Mean serum myostatin concentrations for Phase 1 are shown in Table 1. The low dose ranged from 268 to 420 mg/kg. There was not significant inhibition of serum myostatin concentrations in the low-dose treatment group when compared to the low-dose placebo at any time point. When comparing the means of each individual time point within the low-dose group (Phase 1, Sequence 1A and 1B), several were significantly different. Of those, only the following were consistent with inhibition of myostatin:

**Table 1:** Mean serum myostatin (pg/mL) by time point for low- and high-dose Fortetropin groups compared to high- and low-dose placebo groups.

	**Mean serum myostatin (picogram/mL)**					
**Time point (hours)**	**0**	**12**	**24**	**36**	**48**	**72**
Placebo (6 g)	30,247	33,258	32,685	33,719	30,083	26,509
Fortetropin (6 g)	29,481	35,522	30,271	33,857	29,996	25,802
*p*	0.80	0.49	0.46	0.97	0.98	0.83
Placebo (12 g)	28,512	33,224	30,219	32,986	28,637	27,482
Fortetropin (12 g)	32,213	37,318	34,947	34,253	32,010	32,460
*p*	0.26	0.21	0.15	0.70	0.30	0.13

*The p-value was calculated using Analysis-of-variance (ANOVA) with p < 0.05 deemed significant*.

The mean serum myostatin concentration in the low-dose Fortetropin group at the 72-h time point (25,802 pg/mL) was lower than the low-dose Fortetropin group at the 12-h (*p* = 0.0032), 24-h (*p* = 0.0361), and 36-h (*p* =0.0143) time points and the low-dose placebo group at the 12-h (*p* = 0.0233) and 24-h (*p* = 0.0361) time points.

#### High-Dose Fortetropin Compared to High-Dose Placebo

The mean serum myostatin concentrations for Phase 2 are shown in Table 1. The high dose administered ranged from 536 to 839 mg/kg. When compared to the placebo group, no differences in serum myostatin concentrations in the high-dose treatment group were identified. When the means of each individual time point within Sequence 2A and 2B were compared, there were no differences in mean serum myostatin concentrations.

#### Comparison of Treatment Groups

A comparison of the means of the low and high doses Fortetropin, as well as the respective *p*-values are shown in Table 2. A dose-dependent treatment response was not identified between the low-dose Fortetropin and high-dose Fortetropin groups; however, the mean myostatin concentration was lower in the low-dose treatment group compared to the high-dose treatment group at the 72-h time point (*p* = 0.043).

**Table 2:** Comparison of mean serum myostatin (pg/mL) of low dose treatment (Fortetropin) compared to high-dose treatment for each time point.

	**Mean serum myostatin (picogram/mL)**					
**Time point (hours)**	**0**	**12**	**24**	**36**	**48**	**72**
Fortetropin (6 g)	29,481	35,522	30,271	33,857	29,437	25,802
Fortetropin (12 g)	32,213	37,318	34,947	34,253	32,011	32,460
*p*	0.40	0.58	0.15	0.90	0.54	**0.04**

*The p-value was calculated using Analysis-of-variance (ANOVA) with p < 0.05 deemed significant. Bold represents a statistical significant value*.

When comparing the means of serum myostatin concentration at individual time points between all groups, inhibition of myostatin was appreciated in the low-dose Fortetropin group at the 72-h time point (25,802 pg/mL) compared to the high-dose placebo group at the 12-h (33,224 pg/mL, *p* = 0.024) and 36-h (32,986 pg/mL, *p* = 0.029) time points.

The mean, median, minimum, maximum, and standard deviation of the low and high dose treatment groups compared to low and high dose placebo groups are shown in Tables 3 and 4. A large variation in range and standard deviation between groups and within individual animals was identified in this study. The most variation in the treatment groups within a specific time point occurred in the Fortetropin low-dose (6 g) group at the 12-h time point with a 29,242 pg/mL (sd = 8, 969) difference between the minimum and maximum (Table 3). This variation was also appreciated in the placebo group as well, with the lowest minimum value recorded at 17,416 pg/mL and the highest maximum value recorded at 52,375 pg/mL.

**Table 3:** Phase 1: Low dose Fortetropin (6 g) vs. Placebo: Mean, median, minimum, and maximum serum myostatin concentrations (pg/mL) and corresponding standard deviation.

	**Mean serum myostatin (picogram/mL)**											
**Time point (hours)**	**0**		**12**		**24**		**36**		**48**		**72**	
**Treatment**	**A**	**B**	**A**	**B**	**A**	**B**	**A**	**B**	**A**	**B**	**A**	**B**
Mean	29,481	30,247	35,522	33,258	30,271	32,685	33,857	33,720	29,996	30,084	25,802	26,509
Median	28,560	31,963	33,948	32,774	30,183	32,307	34,471	32,950	29,437	29,760	24,399	24,894
Minimum	21,194	17,416	20,971	19,741	20,617	21,819	24,279	22,366	24,274	19,345	19,816	18,020
Maximum	40,803	38,077	50,213	52,083	39,683	47,747	40,620	53,200	37,278	38,672	37,070	38,964
Standard Deviation	5,224	5,875	8,969	9,272	6,351	6,838	5,938	8,779	3,648	7,150	4,875	7,433

*A, Fortetropin Treatment Group; B, Placebo Group*.

**Table 4:** Phase 2 high dose Fortetropin vs. Placebo: Mean, median, minimum, and maximum serum myostatin concentrations (pg/mL).

	**Mean serum myostatin (picogram/mL)**											
**Time point (hours)**	**0**		**12**		**24**		**36**		**48**		**72**	
**Treatment**	**A**	**B**	**A**	**B**	**A**	**B**	**A**	**B**	**A**	**B**	**A**	**B**
Mean	32,214	28,512	37,319	33,224	34,947	30,219	34,254	32,986	32,011	28,637	32,460	27,483
Median	32,789	28,623	37,237	33,365	32,926	29,166	31,208	32,804	32,618	29,038	33,696	26,650
Minimum	22,956	21,938	25,814	18,139	26,362	19,292	24,992	18,682	21,766	21,458	22,781	20,957
Maximum	41,985	37,694	57,894	52,375	53,469	40,780	48,617	50,502	44,686	37,350	43,634	38,002
Standard Deviation	7,353	5,028	9,551	9,600	8,117	8,316	8,650	9,372	7,608	4,630	6,319	5,180

*A, Fortetropin Treatment Group; B, Placebo Group*.

### Baseline Serum Myostatin

The mean, median, minimum, maximum, and standard deviation of serum myostatin concentrations for time zero (baseline) of all sequences are shown in Table 5. There is almost a 14,000 difference between the highest maximum (41, 985) and the lowest minimum (28, 512).

**Table 5:** Combined mean, median, minimum, and maximum serum myostatin concentration of all groups (picogram/mL) at Time 0 (baseline) in all treatment groups.

**Treatment**	**Fortetropin (A)**		**Placebo (B)**	
**Dose (g)**	**6**	**12**	**6**	**12**
Mean	29,481	32,214	30,247	28,512
Median	28,560	32,786	31,963	28,623
Minimum	21,194	22,956	17,416	21,938
Maximum	40,803	41,985	38,077	37,694
Standard Deviation	5,224	7,353	5,875	5,028

### Effect of Age on Serum Myostatin

The mean age of dogs in the low dose treatment group was 5.0 and the mean age of dogs in the low dose placebo group was 4.2. The mean age of dogs in the high dose Fortetropin and placebo groups was 4.5 and 4.7, respectively. At time zero, there was no dosing effect at either dose of Fortetropin for age, BCS, or bodyweight with *p*-values of 0.94, 0.80, and 0.81, respectively.

The mean serum myostatin concentration in dogs ranging in age from 2 to 4 years was 321,870 pg/mL. The mean serum myostatin concentration in dogs ranging in age from 5 to 8 years was 29,305 pg/mL. The mean serum myostatin concentration in the dogs older than 4 years of age was less than the concentration in dogs 4 years and under (*p* = 0.042).

## Discussion

A single low (6 g) or high dose (12 g) of the oral supplement Fortetropin did not decrease circulating serum myostatin over a 72-h period in healthy adult dogs when compared to placebo. Furthermore, our study did not support the hypothesis that a single dose of Fortetropin would reduce circulating myostatin in healthy dogs. A wide variation in baseline myostatin within groups and individuals over a 72-h period was appreciated in this study. The upregulation of myostatin in response to disease, disuse, or injury may make it a more readily available target for therapeutic inhibition in sick or injured dogs. This theory is supported by the current understanding of muscle hypertrophy and atrophy signaling pathways. The two primary growth factors for these pathways are insulin-like growth factor 1 (IGF-1) and transforming growth factor β, superfamily 8/myostatin. Some crosstalk between pathways has been identified (that is, direct IGF-1 inhibition of myostatin) (15); however, the primary mechanisms are independent of each other (16).

A second aim of this study was to establish normal circulating myostatin values in healthy dogs. Our study revealed a wide variation of serum myostatin concentration between and within individual animals at baseline, and in the treatment and placebo groups. Minimum and maximum values within the treatment groups were as low as 19,816 pg/mL and as high as 57,894 pg/mL, and this variation did not correspond to the administration of the test agent.

The data from our study suggests that there can be significant changes even within the same 24-h period in a relatively homogenous group of animals. Further research is required to determine what influences normal myostatin variation within and between animals. A larger study with age, sex, and body condition score cohorts is needed to establish baseline myostatin in dogs.

Our findings are in contrast with those of the White et al. study, which is the only other study evaluating the effects of Fortetropin on myostatin in dogs (13). In that study, serum myostatin levels were measured in dogs with a cranial cruciate ligament (CCL) injury that was treated with a TPLO. One explanation for the differing results lies in the dosing regimen in each study.

Dogs in the White et al. study received 300 mg/kg of Fortetropin or a nutrient-matched placebo daily for 12 weeks. No changes in serum myostatin were appreciated in the treatment group (*p* > 0.05), whereas dogs in the placebo group experienced an *increase* in myostatin during the first 8 weeks of the study (*p* = 0.02). This suggests that a single dose of Fortetropin does not inhibit serum myostatin but that repeated daily dosing of Fortetropin prevents increased upregulation of myostatin in dogs with a CCL injury treated with a TPLO. The results of the Evans et al., study where older adult men and women who received daily dosing of Fortetropin for 21 days had increased fractional synthetic rate of protein, further supports that a longer dosing regimen is required to achieve a therapeutic effect of Fortetropin (14).

Another possible explanation for the disparate results between our study and the other most recent studies evaluating Fortetropin concern the study populations. The populations tested in the Evans and White studies were either experiencing an orthopedic injury (cranial cruciate ligament rupture and subsequent surgery) or were older and therefore more likely to experience an age-related loss of muscle mass. Furthermore, the White study population was activity restricted for a period of 8 weeks following surgery, which corresponded to the increase in circulating myostatin in the placebo group (*p* = 0.02). Our study tested Fortetropin in adult (non-senior) healthy dogs with no evidence of muscle atrophy or orthopedic disease. Additionally, the dogs in our study were not activity restricted and could continue normal activity throughout the duration of the study.

Circulating serum myostatin levels are upregulated in response to inflammatory cytokines in diseases such as AIDS, cancer, and sepsis (3, 12, 15–19). Myostatin upregulation has also been identified in humans following anterior cruciate ligament injury (20). Given that myostatin is highly preserved across species (9, 15–23), it is reasonable to presume that it is also upregulated in dogs with cranial cruciate ligament injuries. The White et al. study supports the hypothesis that daily dosing of Fortetropin counteracts myostatin upregulation in dogs with a cruciate injury treated with a TPLO.

An age-related increase in myostatin, which as has been documented in older humans, was not appreciated in our study. In fact, the group of older dogs had *lower* myostatin (*p* = 0.0419). Similarly in the Evans study, the 18% increase in fractional synthetic rate of protein synthesis in the treatment group did not correlate to changes in myostatin (14). It is difficult to draw a meaningful conclusion regarding the lack of age-related myostatin decrease in the dogs older than 5 in our study because the population of animals was quite small and because of lack of power analysis, potentially leading to type II error. Furthermore, senior and geriatric animals were excluded from the study. Certainly, the lack of demonstration of an age-related increase in myostatin in our study could be related to the assay used to evaluate myostatin.

Bergen et al. has proposed that low-powered liquid chromatography may be a better method than ELISAs when evaluating changes in myostatin because of its ability to distinguish between latent and active forms of myostatin (11). The same GDF-8/Myostatin Immunoassay Quantikine ELISA kit (R&D Systems, Inc., Minneapolis, Minnesota) was used in our study to evaluate serum myostatin as was used in the White et al. (13) study. Although this assay has been validated across species, including dogs, it may not be the ideal assay to evaluate serum myostatin for some of the same factors highlighted in the Bergen et al. study (17). One possible explanation for this lies in the different forms of myostatin present in the body and its respective receptors.

ActIIBR, the myostatin receptor, has multiple possible ligands that may cause cross-reaction when measuring using an ELISA. Furthermore, the exact mechanism through which Fortetropin suppresses myostatin is proprietary; however, if its primary function is to block intracellular signaling via the ActIIBR receptor, that may explain the lack of inhibition seen after a single dose. Because of the ActIIBR multiple possible ligands, a single antagonist may not specifically block myostatin action. Additionally, the ELISA may not be sensitive enough to distinguish adequately between latent and biologically active myostatin.

As has been recognized in human myostatin research, the ideal assay for assessing circulating myostatin in dogs is not yet established, making evaluation of therapeutics designed to inhibit myostatin challenging. Objective measures such as thigh circumference, total pressure index, and protein synthesis may provide a better assessment of the effect of Fortetropin than circulating serum myostatin (13, 14). Despite these challenges, myostatin remains an important biomarker for dogs, both as a therapeutic target and potentially in further characterizing muscle atrophy and muscle wasting secondary to sarcopenia and cachexia in dogs (2, 11, 13).

This study serves as a starting point for further investigation into the pharmacodynamics and therapeutic dosing regimen of Fortetropin. Oral supplements are not held to the same standards as pharmaceutical drugs regarding safety and efficacy testing; however, they continue to be recommended by practitioners and purchased by the general public. Research to establish dosing regimens, efficacy, safety, and bioavailability of oral supplements drives evidence-based medicine as a standard for incorporating supplements as part of a multimodal treatment of disease.

### Limitations

There are several limitations to this study, the most significant of which is that it only evaluated the effect of a single dose of the two concentrations of Fortetropin on myostatin in healthy dogs. A single dose was chosen based on the unpublished data from the pilot study and to test the proposed mechanism of action. A study with multiple groups comparing different dosing regimens would have been more likely to elucidate a specific dose regimen that would result in myostatin inhibition.

Another limitation is in the population of the study dogs. The enrolled dogs included only one male dog that was intact and the remaining were spayed females. The exact effect, if any, of sex and neuter status on myostatin is unknown. Current literature supports that it is unlikely that the sex status of the dogs in this study significantly affected the outcome (18–20); however, further research is needed to completely understand the effects of sex hormones on circulating myostatin in dogs.

Other limitations include the small study population and that it is not representative of the typical patient population who would be treated with this type of supplement.

## Conclusion

Administration of a single dose of Fortetropin at 6 or 12 g did not inhibit circulating myostatin in healthy adult dogs over a 72-h period. Further research is needed to establish the therapeutic dose and dosing regimen of Fortetropin in healthy, adult dogs. Normal variations of serum myostatin concentration within and between dogs was not established in this study. The best assay to quantify serum myostatin concentrations in dogs has yet to be determined.

Myostatin is an important biomarker in evaluating muscle atrophy. To be a truly useful biomarker, comparison of myostatin to body condition score (BCS), muscle strength, lean body mass, and other biochemical markers should be established in healthy and diseased dogs. Only then can the potential of inhibiting myostatin as a therapeutic target be fully understood and realized.

## Data Availability Statement

The raw data supporting the conclusions of this article will be made available by the authors, without undue reservation.

## Ethics Statement

The animal study was reviewed and approved by Philip Fox, DVM, DACVIM (Cardiology), DACVECC Animal Medical Center. Written informed consent was obtained from the owners for the participation of their animals in this study.

## Author Contributions

CNB and LA were responsible for the hypothesis generation, experimental design and organization, and conduction of the experiments. CNB, LA, and KL were responsible for interpreting and analyzing the results as well as writing and revising the final manuscript. All authors contributed to the article and approved the submitted version.

## Conflict of Interest

Myos Corp provided treatment and placebo samples used in the study and funding to support laboratory testing. KL is the owner of Lamb Consulting. The remaining authors declare that the research was conducted in the absence of any commercial or financial relationships that could be construed as a potential conflict of interest.

## Abbreviations

CCL: Cranial Cruciate Ligament
TGF-β: Transforming growth factor beta
TPLO: Tibial plateau leveling osteotomy
GDF-8: Growth differentiation factor 8 (Myostatin)
ELISA: Enzyme-linked immunoassay
IGF-1: Insuline-like growth factor-1
ActIIBR: Activin IIB Receptor.
